# Inhibition of cyclin-dependent kinase 4 as a potential therapeutic strategy for treatment of synovial sarcoma

**DOI:** 10.1038/s41419-018-0474-4

**Published:** 2018-04-18

**Authors:** Xiaoyang Li, Nicole A. Seebacher, Cassandra Garbutt, Hangzhan Ma, Peng Gao, Tao Xiao, Francis J. Hornicek, Zhenfeng Duan

**Affiliations:** 1 Sarcoma Biology Laboratory, Department of Orthopaedic Surgery, David Geffen School of Medicine at University of Los Angeles, Los Angeles, CA 90095 USA; 2 Department of Orthopedics, The Second Xiangya Hospital of Central South University, Changsha, Hunan 410011 China; 3 Sarcoma Biology Laboratory, Center for Sarcoma and Connective Tissue Oncology, Massachusetts General Hospital and Harvard Medical School, Boston, MA 02114 USA

## Abstract

Synovial sarcoma is a highly aggressive but rare form of soft tissue malignancy that primarily affects the extremities of the arms or legs, for which current chemotherapeutic agents have not been proven to be very effective. The cyclin-dependent kinase 4/6-retinoblastoma protein (CDK4/6-Rb) pathway of cell cycle control is known to be aberrant in a large proportion of cancers. Recently, CDK4 inhibitors have successfully been used pre-clinically for the treatment of many human cancers, and in 2015, following the success of clinical trials, the FDA approved the first selective CDK4/6 inhibitor, palbociclib, for the treatment of endocrine therapy resistant breast cancers. However, the expression and therapeutic potential of targeting CDK4 in synovial sarcoma remains unclear. In the present study, we report that CDK4 is highly expressed in human synovial sarcoma, and high CDK4 expressions are associated with poor prognosis in sarcomas patients and the clinical stage and the TNM grade in synovial sarcoma patients. Knockdown of CDK4 with specific small interference RNAs inhibits cell proliferation and enhances apoptotic effects in synovial sarcoma cells. CDK4 inhibitor palbociclib suppresses synovial sarcoma cell proliferation and growth in a dose and time-dependent manner. Palbociclib also inhibits the CDK4/6-Rb signaling pathway and promotes cell apoptosis without changing CDK4/6 protein levels, suggesting that palbociclib only represses the hyper-activation, not the expression of CDK4/6. Flow cytometry analysis reveals that palbociclib induces G1 cell-cycle arrest and apoptotic effects by targeting the CDK4/6-Rb pathway in synovial sarcoma cells. Furthermore, wound healing assays demonstrate that inhibition of the CDK4/6-Rb pathway by palbociclib significantly decreases synovial sarcoma cell migration in vitro. Our study highlights the importance of the CDK4/6-Rb pathway in human synovial sarcoma pathogenesis, and the role of the current selective CDK4/6 inhibitor, palbociclib, as a potential promising targeted therapeutic agent in the treatment of human synovial sarcoma.

## Introduction

Synovial sarcoma (SS) is a high-grade subtype of soft tissue sarcoma that occurs mainly in children and young adults, characterized by the chromosomal translocation t(X;18) (p11.2; q11.2)^1–3^. The current treatment for localized synovial sarcoma is surgery, occasionally with the combination of additional radiotherapy and chemotherapy, and the published five-year survival rate varies from 40% to 60%^4,5^. However, once the primary disease advances with pulmonary metastasis and relapse, the prognosis is poor, even if under the intensive multi-agent chemotherapy. The limited availability of effective therapeutic measures indicates an urgent clinical need for novel alternative treatment strategies for patients with synovial sarcoma.

Aberrations in cell cycle control is defined as one of the hallmarks of cancer, and may be a favorable target for the improvement of new therapeutic options for the treatment of sarcoma^6,7^. As one of the essential signaling pathways involved in cell cycle progression, the cyclin-dependent kinase (CDK) 4/6-retinoblastoma protein (Rb) pathway (CDK4/6-Rb pathway) is frequently found to be aberrant in cancer^8^. CDK4 is one of the serine/threonine (Ser/Thr) protein kinases that mediates cell cycle progression through the G1-S phase, in preparation for DNA synthesis^9^. The heterodimers formed by CDK4, or its close homolog CDK6, with D-type cyclins (cyclin D) are critical for cell cycle progression. In human malignancies, CDK4 associates with cyclin D and regulates the cell cycle through hyperphosphorylation and deactivation of the tumor suppressor retinoblastoma protein (Rb)^10,11^. Specifically, in response to pro-proliferative stimuli, cyclin D1 associates with CDK4 and gains access to the nuclear cyclin D1-CDK4 complex^12^. These active cyclin D/CDK4 complexes induce the phosphorylation of Rb, and thereby switch off the tumor suppressing function of Rb^13^. The hyperphosphorylated form of Rb is no longer able to bind with the transcription factor E2F1, leading to cancer cell cycle progression through activated transcription of various cell-cycle and anti-apoptotic genes^14,15^. Activation and amplification of the cyclin D/CDK4/Rb pathway has been shown to correlate with uncontrolled tumor cell growth and proliferation in various types of malignancies, including in sarcoma^16^.

CDK4/6 specific inhibitors are the most clinically advanced type of CDK inhibitor, and notably, a dual CDK4/6 inhibitor, palbociclib (IBRANCE^®^). Although palbociclib was initially developed to target the ATP-binding site of CDK4, due to the high homologous and structural similarities between CDK4 and CDK6, palbociclib also targets CDK6. Palbociclib was the first drug in this class to receive Food and Drug Administration (FDA) approval as initial endocrine-based therapy for the treatment of postmenopausal women with hormone receptor (HR)-positive/human epidermal growth factor receptor 2 (HER2)-negative advanced or metastatic breast cancer in combination with an aromatase inhibitor, letrozole, or the selective estrogen receptor downregulator, fulvestrant^17–21^. The FDA have since also approved the CDK4/6 inhibitors, ribociclib (KISQALI®) and abemaciclib (Verzenio^TM^), for a similar application^22^. These agents have also been investigated in other solid tumors, ranging from melanoma to non-small cell lung cancer^23,24^.

Although the field of targeted-therapy for carcinomas is growing rapidly, trials with targeted treatment for rare cancers, such as sarcomas, remain scarce. Interestingly, palbociclib is currently registered in a phase II clinical trial for patients with well-differentiated or dedifferentiated liposarcoma^25,26^. A recent study has reported that nuclear phosphorylated Rb (pRb) expression may be correlated with a poor prognosis for synovial sarcoma patients, and put forward for consideration the potential of palbociclib for patient treatment^27^. However, as a critical part of the Cyclin D/CDK4/6/Rb axis, the expression and clinical significance of CDK4, and the potential mechanism of targeting CDK4 using the specific inhibitor palbociclib as a putative therapeutic strategy, remains to be elucidated in synovial sarcoma. Therefore, in this study, we evaluate the expression of CDK4 and its therapeutic applications in human synovial sarcoma, as well as test palbociclib as a possible treatment for synovial sarcoma.

## Results

### CDK4 is highly expressed in human synovial sarcoma cell lines

**Fig. 1:**
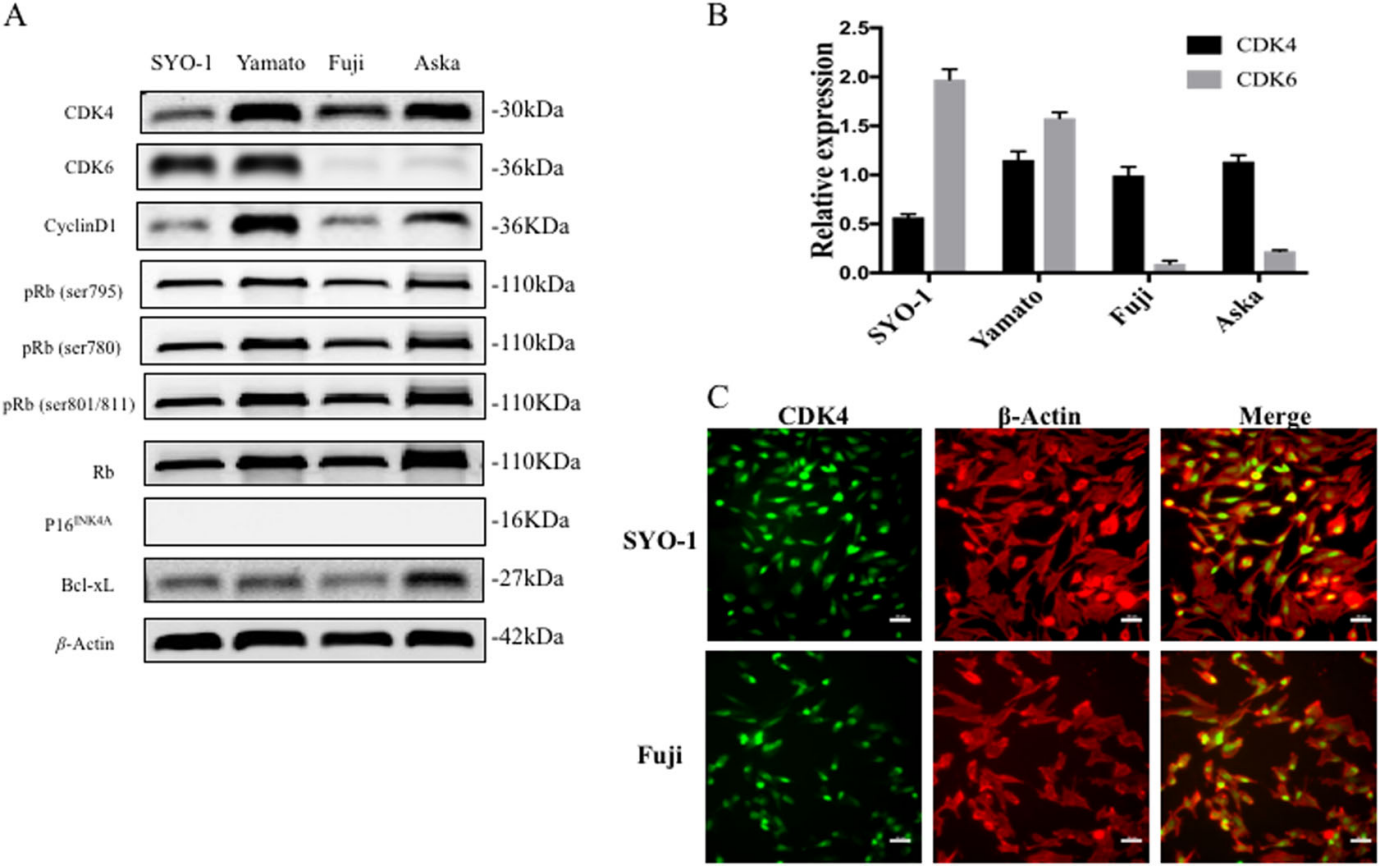
CDK4 is highly expressed in human synovial sarcoma cell lines. **a** Expression levels of CDK4, CDK6, cyclin D1, p16^INK4A^, Rb, pRb (Ser795, 780 and 807/811) and Bcl-xL in four synovial sarcoma cell lines SYO-1, Yamato, Fuji, and Aska were detected using Western blotting. **b** Relative expressions of CDK4 and CDK6 compared with β-actin in different synovial sarcoma cell lines were figured. **c** Expression of CDK4 in SYO-1 and Fuji cells was assessed by immunofluorescence with antibodies to CDK4 and β-Actin. Cells were visualized under a fluorescence microscope after incubation with Alexa Fluor 488 goat anti-rabbit IgG (green) or Alexa Fluor 594 goat anti-mouse IgG (red), and the images were merged (scale bar, 50 μm)

As demonstrated by western blotting, all these human synovial sarcoma cell lines exhibit high levels of CDK4 expression (Fig. 1a). We also assessed the expression of CDK6, cyclin D1, p16^INK4A^, Bcl-xL, Rb and phosphorylated Rb (pRb: Ser795, 780 and 807/811). All of the four synovial sarcoma cell lines also expressed cyclin D1, pRb, Rb, and Bcl-xL (Fig. 1a). In contrast, none of these cell lines expressed p16^INK4A^. CDK6 was highly expressed in the SYO-1 and Yamato cell lines, but was undetectable in the Fuji and Aska cell lines (Fig. 1a). Relative expressions of CDK4 and CDK6 were compared with β-actin in different synovial sarcoma cell lines (Fig. 1b). To further assess the subcellular localization of CDK4 in synovial sarcoma cells, immunofluorescence was performed in SYO-1 and Fuji cells. The green staining represents the CDK4 protein, and the red staining represents β-Actin in the cellular cytoplasm. As shown, CDK4 protein is mostly localized in the nucleus of synovial sarcoma cells, but with some localized to the cytoplasm (Fig. 1c).

### CDK4 expression levels correlates with the clinicopathological characteristics of synovial sarcoma patients

To further validate the clinical significance of CDK4 expression in patients with synovial sarcoma, we tested the expression levels of CDK4 in a human synovial sarcoma tissue microarray (TMA) by immunohistochemistry (IHC), and evaluated the correlation between CDK4 expression and the pathological characteristics of the synovial sarcoma patients. The expression levels of CDK4 were graded based on the distribution (1 < 10%, 2 = 10–25%, 3 = 26–50%, 4 = 51–75%, 5 > 75%) with score ≥ 3 considered high expression.

**Fig. 2:**
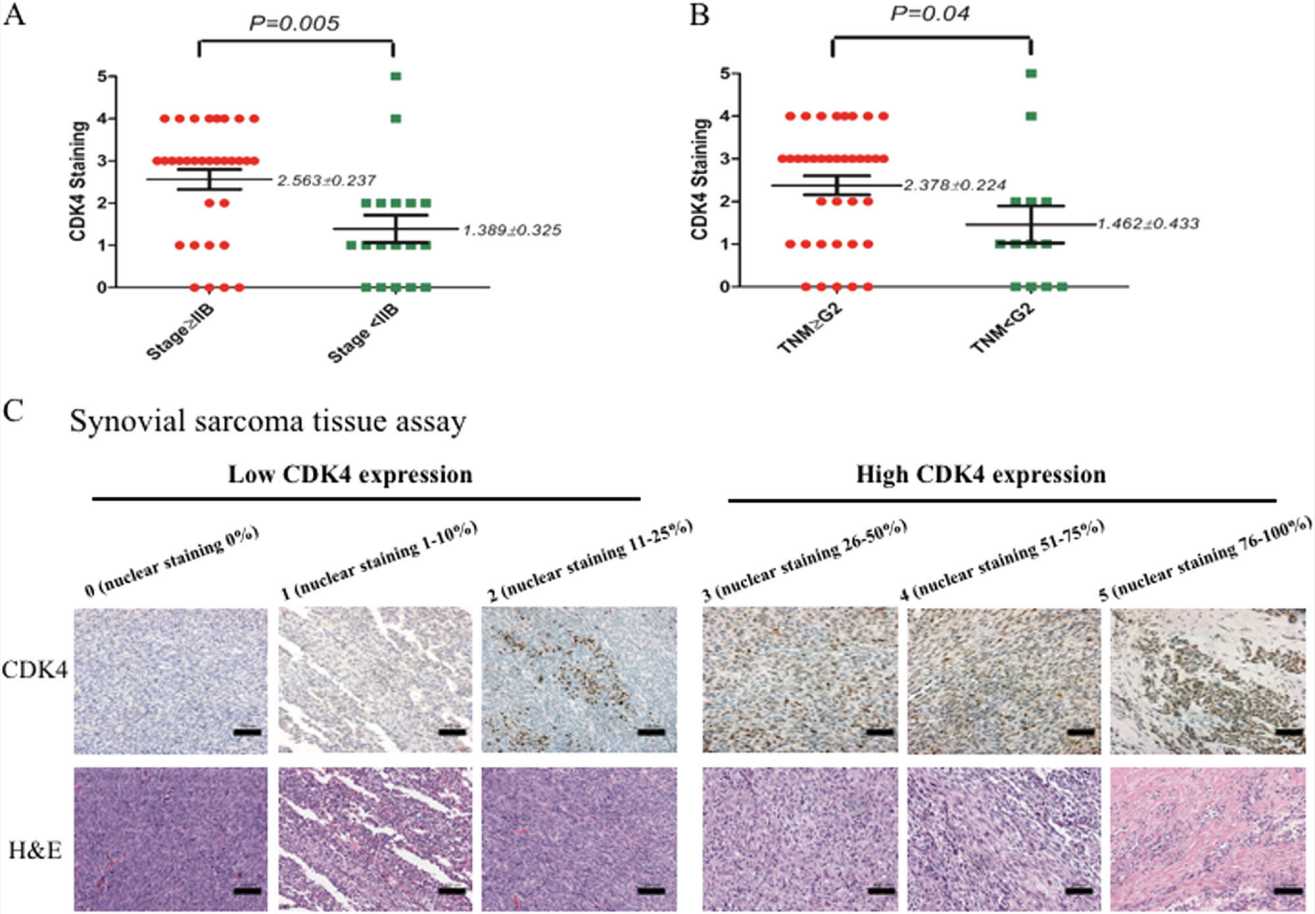
CDK4 expression levels correlate to clinicopathological characteristics of synovial sarcoma patients. CDK4 levels in human synovial sarcoma tissue microarray were determined by immunohistochemistry, and the correlation of CDK4 expression with clinicopathological characteristics of synovial sarcoma patients was evaluated. **a** Distribution of CDK4 staining scores among clinical stage≥IIB and clinical stage<IIB synovial sarcoma tissues. **b** Distribution of CDK4 staining scores in the synovial sarcoma tissues from TNM grade≥G2 patients and TNM grade<G2 patients. **c** Representative images of different immunohistochemical staining intensities of CDK4 ( × 200; scale bar=100 µm). On the basis of the percentage of cells with positive nuclear staining, CDK4 staining patterns were categorized into 6 groups: 0, no nuclear staining; 1+:<10% of positive cells; 2+, 10–25% of positive cells; 3+, 26–50% of positive cells; 4+, 51–75% of positive cells; 5+,>75% of positive cells

The IHC results demonstrate that CDK4 immunoreactivity is in the nucleus of synovial sarcoma tissue cells (Fig. 2c). Of the 50 TMA cases of synovial sarcoma, 41 (82.0%) of the tissues express detectable CDK4 expression. Following evaluation of the clinical history of the human synovial sarcoma specimens, no significant correlations are found between the expression of CDK4 and patient age, gender, tissue type, or tumor location (Supplementary Table 1). However, we find that CDK4 expression significantly correlated with the clinical stage and TNM grade of the synovial sarcoma (both P < 0.05). As shown, CDK4 expression is significantly higher in the higher clinical stages (≥IIB) compared with those in the lower clinical stages (<IIB) (Fig. 2a). Moreover, CDK4 expression is significantly higher in the higher TNM grade (≥G2) tissues, compared to the lower TNM grade (<G2) tissues (Fig. 2b).

**Fig. 3:**
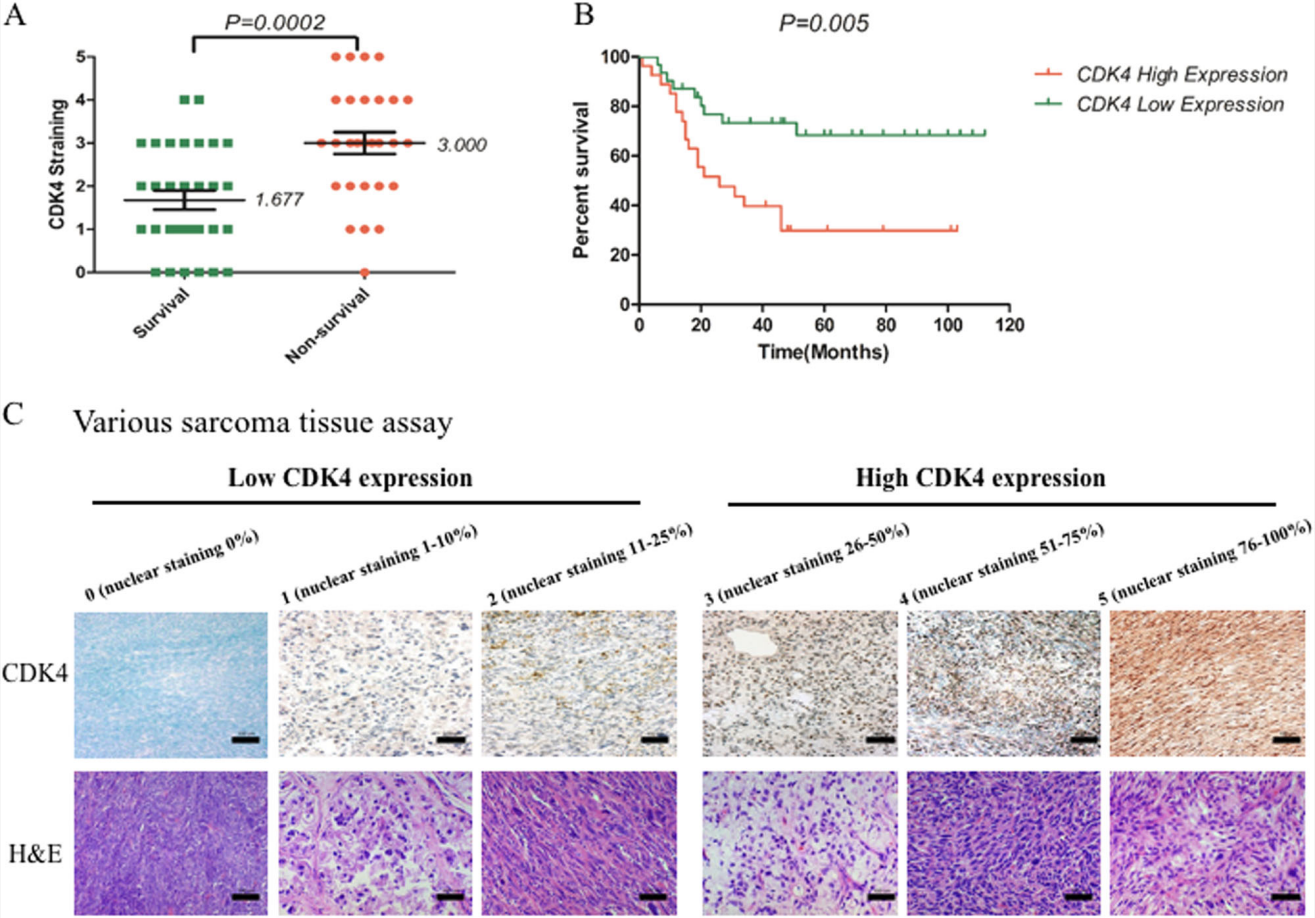
CDK4 expression levels associate with clinicopathological characteristics of various sarcomas patients. CDK4 levels in human various sarcomas tissue microarray were determined by immunohistochemistry, and the relationship between CDK4 expressions and clinicopathological characteristics of various sarcomas patients was analyzed. **a** Distribution of CDK4 staining scores in the synovial sarcoma tissue samples from survival and non-survival patients. **b** Kaplan–Meier survival curve of sarcoma patients with CDK4 high staining (≥3) or low staining (<3). **c** Representative images of different immunohistochemical staining intensities of CDK4 (×200; scale bar:100 µm). On the basis of the percentage of cells with positive nuclear staining, CDK4 staining patterns were categorized into 6 groups: 0, no nuclear staining; 1+:<10% of positive cells; 2+, 10–25% of positive cells; 3+, 26–50% of positive cells; 4+, 51–75% of positive cells; 5+, >75% of positive cells

Considering the absence of information available about the patients’ follow-up and clinical outcomes of the synovial sarcoma TMA, we correlated the CDK4 expression levels to the clinical prognosis of the various types of sarcoma TMA. Various types of sarcoma TMA specimens were also assessed for CDK expression and localization, demonstrating varying degrees of CDK4 staining in the cell nucleus (Fig. 3c). Among the tested 59 sarcoma TMA cases, 52 (88.1%) of the tissues express CDK4. Based on data from up to 112 months of follow-up, CDK4 expression levels in samples from non-survivors are significantly higher than those from survivors (Fig. 3a). Importantly, Kaplan–Meier survival analysis shows that the outcomes for patients in the CDK4 high-staining (≥3) group are worse than for those in the CDK4 low-staining (<3) group (Fig. 3b). In patients with sarcomas, high CDK4 expression is associated with shorter overall survival period vs. low CDK4 expression (P < 0.05). On further analysis, significant correlations are not found between CDK4 expression and sarcoma patient age, gender, sarcoma type, and tumor location (Supplementary Table 2).

### CDK4 silencing with specific siRNAs suppresses cell proliferation and enhances apoptotic effect in synovial sarcoma cell lines

**Fig. 4:**
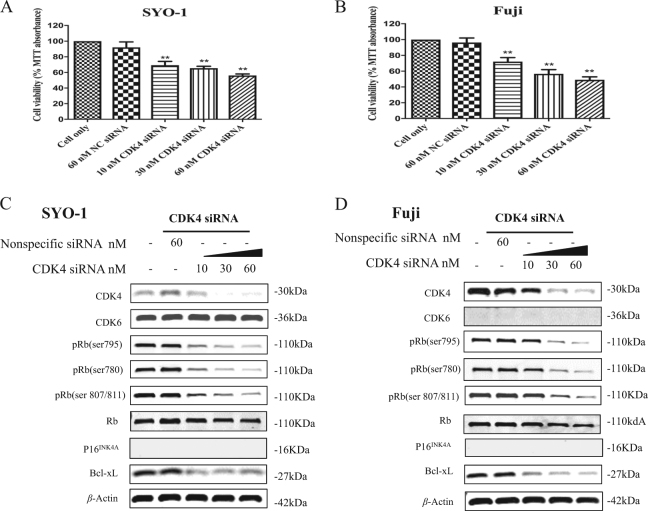
CDK4/6-Rb pathway inhibition induced by CDK4 specific siRNA (#SASI_Hs01_00122488) decreases cell proliferation and promotes apoptosis in synovial sarcoma cells. Human synovial sarcoma SYO-1 and Fuji cells were transfected with increasing concentrations of CDK4 specific siRNA (#SASI_Hs01_00122488) or nonspecific siRNA, and cell proliferation and growth was determined subsequently. **a**, **b** Cell viability was determined by MTT assay after siRNA transfection for 5 days. **c**, **d** The respective proteins of CDK4/6-Rb-apoptosis pathway in cells were examined by Western blotting after 48 h of siRNA transfection. **P *<* 0.01 compared with the cell only group

To validate the effect of CDK4/6-Rb pathway in synovial sarcomas in vitro, we knocked down expression of CDK4 using two CDK4 specific siRNAs. As shown by MTT assays, after transfection with increasing concentrations of CDK4 siRNA #SASI_Hs01_00122488 (Fig. 4a, b) and CDK4 siRNA #SASI_Hs01_00122490 (Suppl. Figures 1A and 1B) for 5 days, the cell viability is dose-dependently inhibited in both SYO-1 and Fuji cells, which is not observed in the nonspecific siRNA transfected cells. Furthermore, western blotting shows that CDK4 siRNA transfection significantly reduces CDK4 expression, whereas the expression of CDK6 remains unchanged. The expression of p16^INK4A^ is undetectable in SYO-1 and Fuji cell lines (Fig. 4c, d, Suppl. Figures 1C and 1D). After transfection with CDK4 siRNA for 48 h, the expression of pRb is reduced in a dose-dependent manner, whereas the Rb expression is not significantly changed (Fig. 4c, d, Suppl. Figures 1C and 1D). A dose-dependent decrease in the expression of the anti-apoptotic protein, Bcl-xL, is also observed after transfection with CDK4 siRNA (Fig. 4c, d, Suppl. Figures 1C and 1D).

### Palbociclib inhibits cell proliferation and promotes apoptosis by targeting the CDK4/6-Rb pathway in human synovial sarcoma cells

**Fig. 5:**
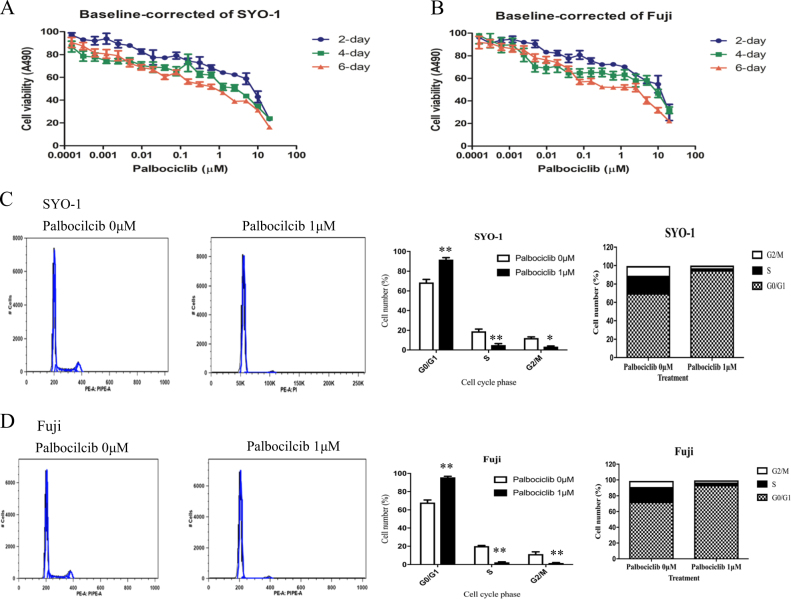
Inhibition of CDK4/6-Rb pathway by palbociclib decreases cell proliferation and induces cell cycle arrest in human synovial sarcomas. Human synovial sarcoma SYO-1 and Fuji cells were treated with increasing concentrations of palbociclib for the indicated time, and cell proliferation and growth was determined subsequently. **a**, **b** Cell viability was determined by MTT assays after palbociclib exposure for 2, 4, and 6 days. The cell cycle of SYO-1 and Fuji cells was assessed after exposure to palbociclib (1 µM) for 24 h by flow cytometry analysis. Representative images of cell cycle distribution in SYO-1 (**c**) and Fuji (**d**) cells with or without palbociclib treatment. Cell numbers in different cell cycle phases were counted. Different cell cycle phase rates were also analyzed. *P *<* 0.05, **P *<* 0.01 compared with the cell only group

**Fig. 6:**
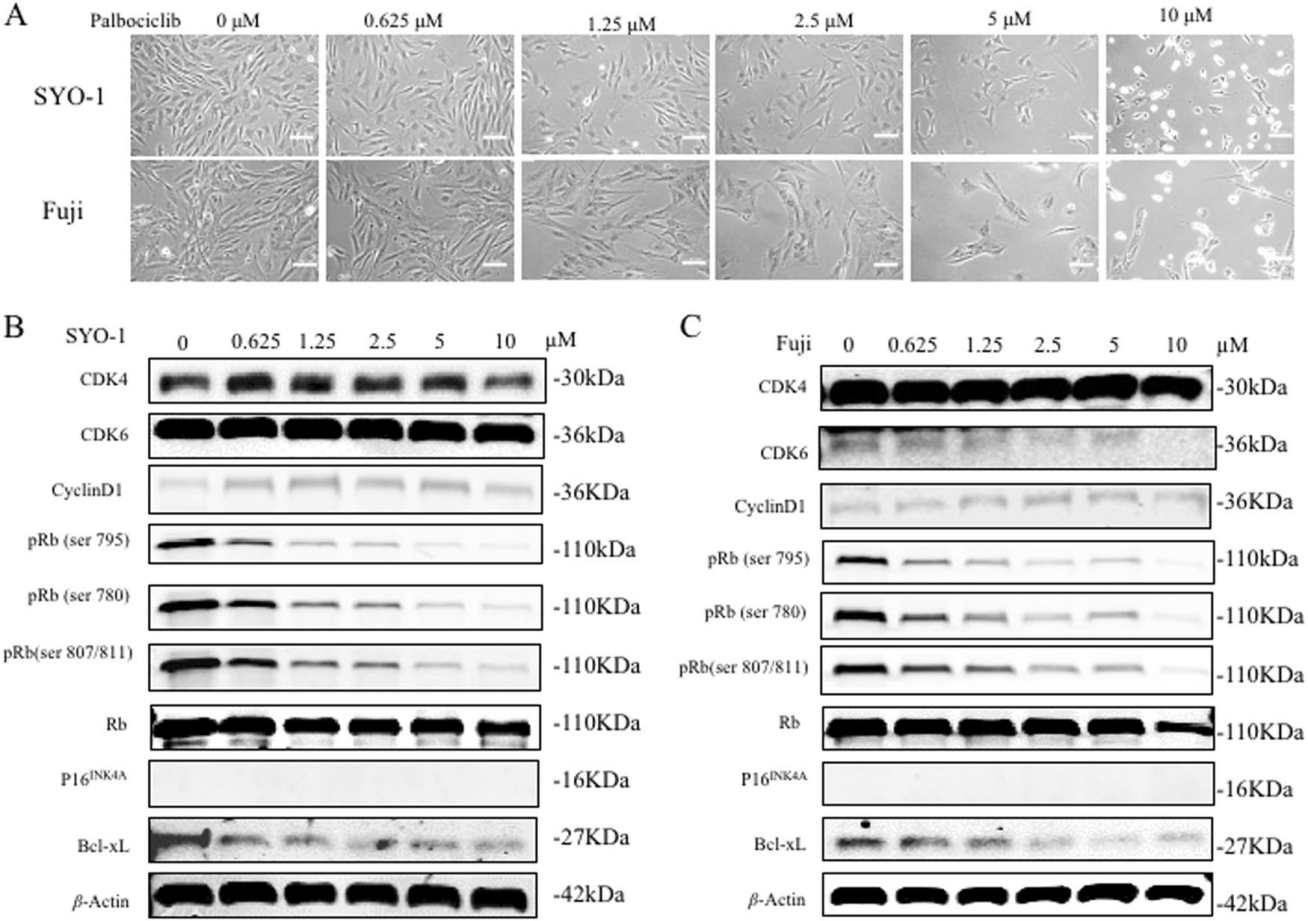
Inhibition of CDK4/6-Rb pathway by palbociclib alters phosphorylation of Rb and promotes apoptosis in human synovial sarcomas. **a** Human synovial sarcoma SYO-1 and Fuji cells were treated with increasing concentrations of palbociclib for the indicated time, and the morphologic changes of cells were observed by microscopy after 2 days of palbociclib treatment (scale bar, 50 μm). The expression of respective proteins in CDK4/6-Rb pathway and apoptosis in SYO-1 (**b**) and Fuji (**c**) cells was examined by western blotting after 3 days of palbociclib treatment

Cell proliferation and growth were determined by MTT assays in synovial sarcoma SYO-1 and Fuji cells with palbociclib treatment. After exposure to increasing concentrations of palbociclib for 2, 4, and 6 days, the cell viability is decreased in a dose-dependent manner in both SYO-1 (Fig. 5a) and Fuji cells (Fig. 5b). Morphologic changes are also observed after 2 days of palbociclib exposure. When treated with increased doses of palbociclib (0, 0.625, 1.25, 2.5, 5, 10 μM) for 48 h, SYO-1 and Fuji cells show pronounced morphologic signs of toxicity, including abnormal shape and appearance, cellular lysis, and destruction. The cells begin to show observable signs of toxicity in the form of cells reduction and abnormal cell morphology when treated with 1.25 μM palbociclib, and cellular death and other abnormalities become more pronounced as the drug concentration continued to increase up to 10 μM (Fig. 6a).

As shown by western blotting analysis, 2 days of palbociclib exposure significantly decreases the expression of pRb (Ser795, 780 and 807/811) in a dose-dependent manner, whereas the expression of Rb is not significantly changed (Fig. 6b, c). All of the cell line samples show no p16^INK4A^ expression. A dose-dependent decrease in the expression of the cell anti-apoptotic protein, Bcl-xL, is also observed with palbociclib treatment. Notably, western blotting shows that the activity of palbociclib had no influence on the expression of CDK4, CDK6 or cyclin D1, suggesting that palbociclib only suppresses the activity of CDK4, but does not change the protein expression (Fig. 6b, c).

### Inhibition of CDK4/6-Rb pathway with palbociclib induces human synovial sarcoma cell cycle arrest and cell apoptosis

Due to high concentrations of palbociclib resulting in pronounced cell death, we used a 1 µM for assessing the cell cycle. After 1 µM of palbociclib treatment for 24 h, a significant G1 cell-cycle arrest accompanied by a reduction in the fraction of cells in S phase is observed in both SYO-1 and Fuji cells (both *P* < 0.05), compared with the non-treated control (Fig. 5c, d). This suggests that CDK4/6-Rb inhibition, induced by palbociclib, is able to promote G1 cell-cycle arrest and inhibit DNA synthesis in synovial sarcoma cells. The cell apoptosis analysis demonstrates increased apoptosis rates in both SYO-1 and Fuji cells after palbociclib exposure for 24 h, as compared with the control group (Suppl. Figures 2A and 2B). Collectively, these results indicate that reduced synovial sarcoma cell proliferation by inhibition of CDK4/6-Rb pathway is associated with induction of cell cycle arrest and cell apoptosis.

### Inhibition of the CDK4/6-Rb pathway with palbociclib reduces human synovial sarcoma cell migration in vitro

**Fig. 7:**
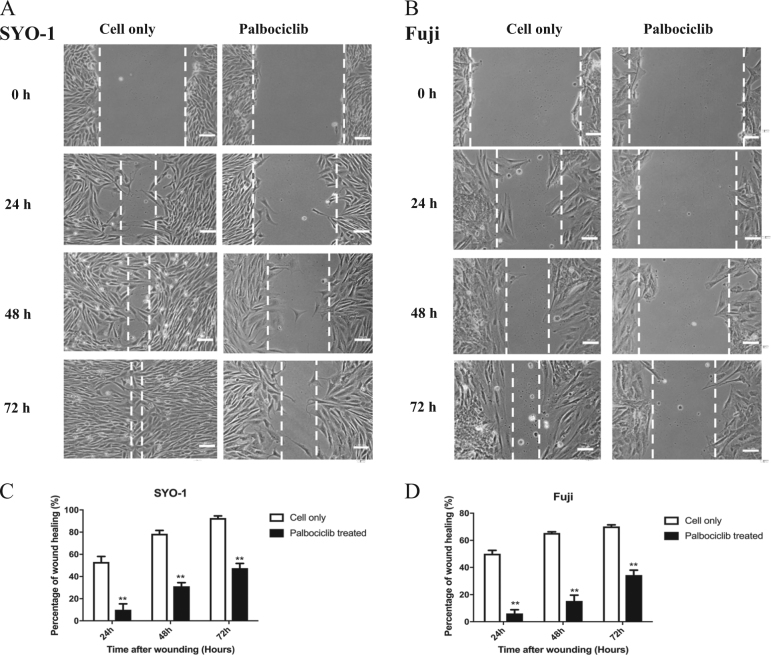
Inhibition of the CDK4/6-Rb pathway by palbociclib reduced human synovial sarcoma cell migration in vitro. After exposure to 1 µM of palbociclib for the indicated time, the cell migration of SYO-1 and Fuji cells was determined by wound healing assays. **a** Representative images of SYO-1 cell migration after palbociclib treatment for 24, 48, and 72 h (scale bar, 50 μm). **b** Representative images of Fuji cell migration after palbociclib treatment for 24, 48, and 72 h (scale bar, 50 μm). **c** Cell migration distance of SYO-1 cells was measured after palbociclib treatment. **d** Cell migration distance of Fuji cells was measured after palbociclib treatment. ***P *<* 0.001 compared with the cell only group

The wound healing assay was performed in SYO-1 and Fuji cells after palbociclib treatment. After exposure to 1 µM of palbociclib for 24, 48, and 72 h, the cell migration activities are significantly and time-dependently suppressed in both SYO-1 and Fuji cells, as compared with the palbociclib-free control groups (*P* *<* 0.01) (Fig. 7).

## Discussion

The CDK4/6-Rb pathway is a fundamental driver for cell cycle control and regulation of cellular apoptosis^28,29^, and could serve as a promising therapeutic target in various human malignancies^30–34^. Since its activity plays a crucial role in the proliferation and progression of cancer, pharmacological inhibitors targeting the CDK4/6 pathway have emerged as an attractive approach in oncology^35,36^. In the current study, we explore the expression and function of the CDK4/6-Rb pathway in synovial sarcomas, and investigate the therapeutic potential of the selective CDK4 inhibitor, palbociclib, in vitro.

We first analyzed the expression levels of CDK4 in synovial sarcoma cell lines and found that CDK4 was highly expressed in all the human synovial sarcoma cell lines, and it was found to be localized to the nucleus by immunofluorescence staining. While previous studies have assessed the expression of CDK4 in some sarcoma types, including liposarcoma and Ewing’s sarcoma^37,38^, none of these studies have assessed the expression of CDK4 in synovial sarcoma. A study by Vlenterie et al, 2016, has analyzed the expression of cyclin D1 and nuclear phosphor-Rb in 43 synovial sarcoma tissue samples, however, this study did not characterize the relationship between the level of CDK4 expression and the clinical characteristics of the patients^27^. In our study, synovial sarcoma TMAs determined by IHC showed that CDK4 expression was observed in most of the tested sarcoma tissue samples. This suggests CDK4 might have an important role in the pathogenesis of synovial sarcoma. Further studies explored the relationship between CDK4 expression and clinicopathological characteristics using a TMA containing 50 synovial sarcoma cases with clinical information, and a TMA containing 59 various sarcoma cases with up to 112 months of follow-up data. Our results showed that CDK4 expression significantly correlated with a higher clinical stage and a higher TNM grade of synovial sarcoma patients, and a worse clinical prognosis of sarcoma patients. A previous study of liposarcoma supports that the overexpression of CDK4 is correlated to a poorer prognosis of patients^39^. A knockdown of CDK4 in fusion-gene positive rhabdomyosarcoma cells has also been shown to abrogate transformation and proliferation *via* G1-phase cell-cycle arrest, demonstrating that CDK4 is also essential for sarcoma cell survival and growth^40^. More recently, amplification and over-activation of the CDK4/Rb pathway has been found in another sarcoma type, chordoma^41^. Altogether, these results verify the expression of CDK4 in synovial sarcomas and highlight its important role in sarcomas, including synovial sarcoma.

In contrast to the previous study that only investigated cyclin D1 and pRb levels following palbociclib treatment^27^, we specifically knocked down CDK4 expression with CDK4 siRNAs to investigate the role of CDK4 in synovial sarcoma cell growth and proliferation. The result showed that CDK4 inhibition by siRNA decreased synovial sarcoma cell proliferation and growth in a dose-dependent manner. We further suppressed the expression of CDK4 in synovial sarcoma cells using the clinically approved CDK4 inhibitor, palbociclib, and explored changes to the cellular phenotypic. Consistently, our findings demonstrated that CDK4 inhibition by palbociclib reduced synovial sarcoma cell proliferation and growth dose-dependently. We demonstrated that palbociclib was acting through inhibition of CDK4/6 activity, not by reducing protein expression. These findings collectively suggest that CDK4 plays a crucial role in promoting synovial sarcoma proliferation and growth.

As a fundamental cell division modulator, CDK4 functions mainly through activation, by phosphorylation, of the retinoblastoma tumor suppressor, inducing G1 cell cycle arrest in tumor cells^42^. In response to mitogenic signals, CDK4 combines with cyclin D1 and transfers into the nucleus to form the active cyclin D1–CDK4 complex and then phosphorylates Rb into pRb. Our immunofluorescence straining showed that CDK4 expression was observed both in the cytoplasm and in the nucleus of SYO-1 and Fuji cells, but mostly localized in the nucleus. It has been suggested that in proliferating cells, during G1 phase, cyclin D1 accumulates in the nucleus and triggers the active cyclin D1-CDK4 complex formation^43^. This leads to release of the E2F transcription factor and activation of downstream target genes, promoting cell cycle progression and ultimately cell proliferation and growth^44^. Notably, the cyclin D/CDK4/Rb pathway is universally disrupted in human cancer^45,46^. Thus, we explored changes occurring in proteins of the cyclin D/CDK4/Rb pathway after CDK4 inhibition. We found that both the CDK4 specific siRNA and palbociclib suppressed downstream Rb phosphorylation and also reduced expression of the cell-survival protein Bcl-xL, in a dose-dependent manner. Bcl-xL is a member in Bcl-2 family, which is known to suppress downstream activators of apoptosis^47^. Interestingly, synovial sarcomas are known to have high levels of Bcl-2 compared to other soft tissue sarcomas^48^. Notably, while palbociclib acted only to inhibit CDK4 activity, with no changes to protein expression, it also effectively downregulated pRb and Bcl-xL expression.

To demonstrate the potential mechanisms underlying CDK4 inhibition caused by palbociclib treatment on synovial sarcoma cell growth, flow cytometry analysis was used to assess the cell cycle and apoptosis in human synovial sarcoma cells following treatment with palbociclib. The results revealed that CDK4 inhibition dramatically induced cell cycle arrest in G1 phase in synovial sarcoma cells after palbociclib treatment. Cell apoptosis determination simultaneously revealed that CDK4 inhibition dramatically induced cell apoptosis in synovial sarcoma cells. Hence, these findings confirmed that CDK4 inhibition, triggered by palbociclib, could decrease synovial sarcoma cell proliferation and growth *via* induction of cell apoptosis via arresting the cell cycle in G1 phase.

p16^INK4A^, encoded by cyclin-dependent kinase inhibitor 2A (CDKN2A) tumor suppressor gene, is one of the first genes noted to be silenced epigenetically in human cancers^49^. Specific somatic loss of p16^INK4A^ through point mutations or small deletions, and silencing of p16^INK4A^ through promoter methylation, have been reported at high frequency in many human cancers^50,51^. Restriction of cell cycle progression occurs through inhibition of the activity of CDK4 by p16^INK4A^ ^37^. A deletion of p16^INK4A^ causes altered activity of CDK4, leading to hyperphosphorylation of Rb and aberrant regulation of the cell cycle^52,53^. Preclinical data indicates that cancers with a loss in p16^INK4A^ may still be sensitive to CDK4 inhibition^54,55^. Palbociclib has been shown to inhibit the growth of doxorubicin-resistant Ewing’s sarcoma patient-derived orthotopic xenografts in vivo, with a p16^INK4A^-deletion^56^. Specific orthotopic mouse models developed from sarcoma patients, may help in the development of individualized therapeutics to precisely target patient specific sarcomas^57–61^. In synovial sarcoma, the deletion of p16^INK4A^ is also a frequent genetic event^62^. This study shows that synovial sarcoma cells, with a loss in P16^INK4A,^ are sensitive to palbociclib treatment, indicating that palbociclib may be a potential therapeutic drug to treat synovial sarcoma.

The development of tumor cell invasion and metastasis is, largely, responsible for the mortality and morbidity of cancer^63^. TMA analysis showed that the overexpression of CDK4 was related to higher clinical stages and higher TNM grades in synovial sarcomas and this correlated with a poorer patient prognosis in various sarcomas, indicating that CDK4 might be necessary for tumor metastasis. The course of metastasis from the primary tumor sites to distant other organs is of principal importance in the prognosis of cancer patients^63^. In synovial sarcoma, a high incidence of late metastasis occurs in about 50% of all cases, which significantly impacts the mortality and reduces patient survival outcomes^64,65^. We then further analyzed the effect of CDK4 inhibition on synovial sarcoma cell migration in vitro. Synovial sarcoma cells treated with non-lethal doses of palbociclib unveiled that CDK4 inhibition reduced synovial sarcoma cell migration, suggesting that CDK4 might be a promotor to synovial sarcoma metastasis, which is the main obstacle in the treatment of synovial sarcoma.

Taken together, our current study demonstrates that CDK4 is highly expressed in synovial sarcomas. Overexpression of CDK4 correlates with higher clinical stages and higher TNM grades in synovial sarcomas and poor survival outcomes of sarcoma patients. CDK4 inhibition decreases synovial sarcoma cell proliferation and growth through apoptosis induction via cell cycle arrest. These findings, together with the current clinical application of palbociclib for other malignancies, highlight CDK4 as a potential therapeutic target and palbociclib as a promising anti-cancer agent for synovial sarcoma treatment.

## Materials and methods

### Cell lines and cell culture

The human synovial sarcoma cell lines SYO-1 and Fuji were kindly provided by Dr. Akira Kawai (National Cancer Center Hospital, Tokyo, Japan) and Dr. Kazuo Nagashima (Hokkaido University School of Medicine, Hokkaido, Japan), respectively^66,67^. The human synovial sarcoma cell lines Yamato-SS and Aska-SS were generously provided by Dr. Kazuyuki Itoh (Osaka Medical Center for Cancer and Cardiovascular Diseases, Osaka, Japan)^68^. All synovial sarcoma cell lines were cultured in RPMI-1640 (Life Technologies, Grand Island, NY) complete media supplemented with 10% fetal bovine serum (GIBCO, Grand Island, NY, USA), 100 U/mL penicillin G, and 100 μg/mL streptomycin (Life Technologies, Carlsbad, CA). All cells were incubated in a humidified 5% CO_2_-95% air atmosphere at 37 °C.

### Immunofluorescence assay

For immunostaining of cultured synovial sarcoma cells, SYO-1 and Fuji cells were grown in 6-well plates for 2 days and fixed with 3.7% paraformaldehyde for 15 min, followed by permeabilization with ice-cold methanol and blocked with 1% bovine serum albumin (BSA). The cells were then incubated with the CDK4 primary antibody (1:200 dilution, #12790, Cell Signaling Technology, Beverly, MA, USA) and β-Actin (1:200 dilution, #sc-47778, Santa Cruz Biotechnology, Dallas, TX, USA) at 4 °C overnight, followed by incubation with Alexa Fluor 488 (Green) conjugated goat anti-rabbit antibody and Alexa Fluor 594 (Red) goat anti-mouse antibody (Invitrogen, Carlsbad, CA, USA) for 1 h. Finally, cells were imaged on a Nikon Eclipse Ti-U fluorescence microscope (Diagnostic Instruments Inc., NY, USA) equipped with a SPOT RT^™^ digital camera.

### Immunohistochemistry analysis of human tissue microarray (TMA)

The expression of CDK4 in the human tissue microarray was determined by immunohistochemistry assay (Paraffin) according to the manufacturer’s instructions (Cell Signaling Technology, Beverly, MA, USA). The synovial sarcoma TMA (US Biomax, Inc., Rockville, MD, USA) contains 50 cases/100 cores with histopathologic data, including age, gender, tissue type, tumor location, clinical stage, TNM grade, and pathology type. The synovial sarcoma TMA information is available at the website (http://www.biomax.us/tissue-arrays/Soft_Tissue/SS1001). The various sarcomas TMAs were purchased from SuperBioChips Laboratories (Seoul, South Korea) and contain 59 paraffin-embedded tumor specimens from 59 individual sarcoma patients. Clinicopathological information including age, gender, sarcoma type, tumor location, the follow-up time and prognostic outcome can be obtained from the website (http://www.tissue-array.com). Hematoxylin and Eosin stained tissue array slides were made available online by the manufacturers.

Briefly, the two 5 μm paraffin-embedded TMA slides were baked for 1 h at 60 °C, deparaffinized in xylene three times for 10 min each, and then transferred through graded ethanol (100 and 95%) twice for rehydration, 10 min each. After heat-induced epitope retrieval, the endogenous peroxidase was quenched by 3% hydrogen peroxide. Following blocking by normal goat serum for 1 h at room temperature, the two slides were incubated with rabbit polyclonal antibody to human CDK4 (#12790, 1:500 dilution, Cell Signaling Technology, Beverly, MA, USA) at 4 °C overnight in a humidified chamber. Subsequently, bound antibody on the array was detected by SignalStain® Boost Detection Reagent (Cell Signaling Technology, Beverly, MA, USA) and SignalStain® DAB (Cell Signaling Technology, Beverly, MA, USA). The nuclei of synovial sarcoma cells were counterstained with hematoxylin QS (Vector Laboratories, Burlingame, CA, USA) to improve images. Finally, the section was mounted with VectaMount AQ (Vector Laboratories, Burlingame, CA, USA) for long-term preservation. The TMA slides were also stained in the absence of CDK4 antibody to evaluate nonspecific secondary antibody reactions. The slide was imaged using an Olympus microscope (BX51, Olympus, PA, USA).

Immunostaining of the whole slide areas were viewed and scored separately by three independent pathologists who were blinded to tumor characteristics and core case details. The expression level of CDK4 was evaluated according to the percentage of cells with positive nuclear staining. Staining patterns were categorized on a semi-quantitative scale from 0–5 + , as follows: 0, no nuclear staining; 1 + , < 10% of positive cells; 2 + , 10–25% of positive cells; 3 + , 26–50% of positive cells; 4 + , 51–75% of positive cells; 5 + , > 75% of positive cells. Tumors with a staining score of ≥ 3 were designated as high CDK4 expression and < 3 were designated as low CDK4 expression.

### Synthetic CDK4 siRNA transfection and drug treatment

CDK4 knockdown was performed by CDK4 specific siRNA transfection in synovial sarcoma cells. The human nonspecific siRNA and CDK4 siRNAs (#SASI_Hs01_00122488, NM_000075.2, 5′-CUCUUAUCUACAUAAGGAU-3′ and #SASI_Hs01_00122490, NM_000075.2, 5′-CACUUACACCCGUGGUUGU-3′) were purchased from Sigma-Aldrich (St. Louis, MO, USA). The nonspecific siRNA oligonucleotides were used as negative controls. Increasing concentrations (0, 10, 30, and 60 nM) of CDK4 siRNAs or nonspecific siRNA (60 nM) were transfected into cells using Lipofectamine^®^ RNAiMAX Reagent (Invitrogen, Carlsbad, CA, USA) according to the manufacturer’s instructions. After 48 h for western blotting or 5 days for MTT assay, transfected cells were subjected to subsequent analysis.

CDK4 inhibition in synovial sarcoma cells was carried out by palbociclib treatment. Palbociclib (PD-0332991) HCl (#S1116, Selleck Chemicals, Houston, TX, USA) is a highly selective inhibitor of CDK4/6 activity. SYO-1 and Fuji cells were grown on 96-well plates for cell proliferation assays, 6-well plates for wound healing assays, or 12-well plates for western blotting analysis, and incubated with various concentrations of palbociclib, followed by subsequent experiments.

### Cell proliferation assay

SYO-1 and Fuji cells were seeded into 96-well plates at a density of 4 × 10^3^ cells per well, and treatments were performed for 5 days after CDK4 siRNA transfection or 2, 4, and 6 days after palbociclib treatment, the cell viability of SYO-1 and Fuji cells was determined using MTT assays. Briefly, at the end of cell treatment, 20 μL of MTT (5 mg/mL, Sigma-Aldrich, St. Louis, MO, USA) was added to each well and the 96-well plates were incubated at 37 °C in a 5% CO_2_-95% air humidified atmosphere for 4 h. Finally, the resulting formazan product was dissolved with 100 μL of acid isopropanol and the absorbance at a wavelength of 490 nm (A490) was measured on a SpectraMax Microplate® Spectrophotometer (Molecular Devices LLC, Sunnyvale, CA, USA). In all experiments, the MTT assays were conducted in triplicate. Meanwhile, light microscope images of the morphological changes of the SYO-1 and Fuji cells were obtained by a Zeiss microscope (Carl Zeiss, Inc., Oberkochen, Germany) with an attached Nikon D40 digital camera (Diagnostic Instruments Inc., NY, USA) equipped with a Zen Imaging software after increasing concentrations (0, 0.625, 1.25, 2.5, 5, 10 µM) of palbociclib treatment for 48 h.

### Protein preparation and Western blotting

Protein lysates were extracted from SYO-1 and Fuji cells with 1× RIPA lysis buffer (Upstate Biotechnology, Charlottesville, VA, USA) supplemented with complete protease inhibitor cocktail tablets (Roche Applied Science, Indianapolis, IN, USA). The concentrations of the protein lysates were determined by DC^TM^ protein assay reagents (BIO-RAD, Hercules, CA, USA) with a Beckman spectrophotometer (Beckman Instruments, Inc., Indianapolis, IN, USA). Equal concentrations of denatured proteins were separated by NuPAGE^®^ 4–12% Bis-Tris Gel (Invitrogen, Carlsbad, CA, USA), and then transferred to nitrocellulose membranes (BIO-RAD, Hercules, CA, USA). After blocking with 5% non-fat milk for 1 h, the membranes were incubated with rabbit monoclonal antibodies to human CDK4, CDK6, cyclin D1, p16^INK4A^, pRb (Ser795, 780 and 807/811), and Bcl-xL (1:1000 dilution, Cell Signaling Technology, Beverly, MA, USA), mouse monoclonal antibodies to human Rb (1:1000 dilution, Cell Signaling Technology, Beverly, MA, USA) and β-actin (1:2000 dilution, Santa Cruz Biotechnology, Dallas, TX, USA) at 4 °C overnight. Following primary antibody incubation, the membranes were washed with TBST for 3 times, 5 min every time, and Goat anti-rabbit IRDye® 800CW (926-32211, 1:5000 dilution, Li-COR Biosciences, NE, USA) or Goat anti-mouse IRDye® 680LT secondary antibody (926-68020, 1:10000 dilution, Li-COR Biosciences, NE, USA) were added, respectively. After incubation at room temperature for 2 h, the bands were detected using Odyssey Infrared Fluorescent Western Blots Imaging System from Li-COR Bioscience (Lincoln, NE, USA). Quantification of Western blotting results was analyzed using Odyssey software 3.0 (Li-COR Bioscience, Lincoln, NE, USA).

### Flow cytometry analysis

The cell cycle status of SYO-1 and Fuji cells were analyzed by flow cytometry after 24 h of palbociclib treatment. SYO-1 and Fuji cells were collected and fixed in 70% ethanol at 4 °C overnight, followed by incubation with RNase A (100ug/mL, Thermo Scientific, Waltham, MA, USA) at 37 °C for 30 min and stained with Propidium Iodide (50ug/mL, Sigma-Aldrich, St. Louis, MO, USA) for an additional 30 min. The DNA content was determined by flow cytometry (FACSCanto II, BD, NJ, USA) and the population of cells in each phase of the cell cycle analyzed with the MultiCycle software (Phoenix Flow Systems, CA, USA).

Cell apoptosis in SYO-1 and Fuji cells were analyzed by flow cytometry after 24 h of palbociclib treatment. For cell apoptosis analysis, the cells were collected by trypsinization and resuspended in annexin-binding buffer, followed by staining with FITC annexin V and Propidium Iodide (Invitrogen, NY, USA) for 30 min, and then subjected to flow cytometry analysis.

### Cell migration assay

Cell migration activity was detected by wound healing assays. SYO-1 and Fuji cells were seeded into 6-well plates at a density of 4 × 10^5^ cells per well and incubated overnight. After cells reached 100% confluency, the adherent cell layer was wounded by scraping three parallel lines with a sterile 10 μL tip, and 1 µM of palbociclib was immediately added into the cell medium for an additional starved incubation with low-serum medium containing 2% FBS, since serum starving is the most common non-pharmaceutical method for minimizing proliferation in wound healing assays^69,70^. Wounds were observed at 0, 24, 48 and 72 h after palbociclib treatment. The wounded cells within each well were photographed each time point under a Zeiss microscope (Carl Zeiss, Inc., Oberkochen, Germany) with an attached Nikon D40 digital camera (Diagnostic Instruments Inc., NY, USA) equipped with Nikon Camera Control Pro 2 Imaging software (Diagnostic Instruments Inc., NY, USA). The wound width was evaluated by measuring the distance between the two edges of the scratch at five sites in each image. Cell migration was determined using the following formula: Percentage of wound healing (%)=(wound width at the 0 h time point−wound width at the observed time point) / wound width at the 0 h time point × 100%.

### Statistical analysis

Statistical analysis was performed using the GraphPad PRISM 5 software (GraphPad Software, San Diego, CA, USA). Data are expressed as mean±SD. Student’s *t*-test was used to determine the statistical significance of differences between groups. Survival analysis was assessed using the Kaplan-Meier method, and significance was determined by the log-rank test. A *P* value of ≤0.05 was considered statistically significant.

## Electronic supplementary material

Suppl. Fig. 1(TIF 1522 kb)

Suppl. Fig. 2(TIF 1522 kb)

Suppl. Table 1(DOCX 70 kb)

Suppl. Table 2(DOCX 79 kb)

Supplementary figure legends(DOCX 79 kb)
