# Supplementary figures and images for: Inhibition of cyclin-dependent kinase 4 as a potential therapeutic strategy for treatment of synovial sarcoma
Source: Cell Death Dis. 2018 Apr 18;9(5):446. doi: 10.1038/s41419-018-0474-4 (PMC5906661; doi:10.1038/s41419-018-0474-4)

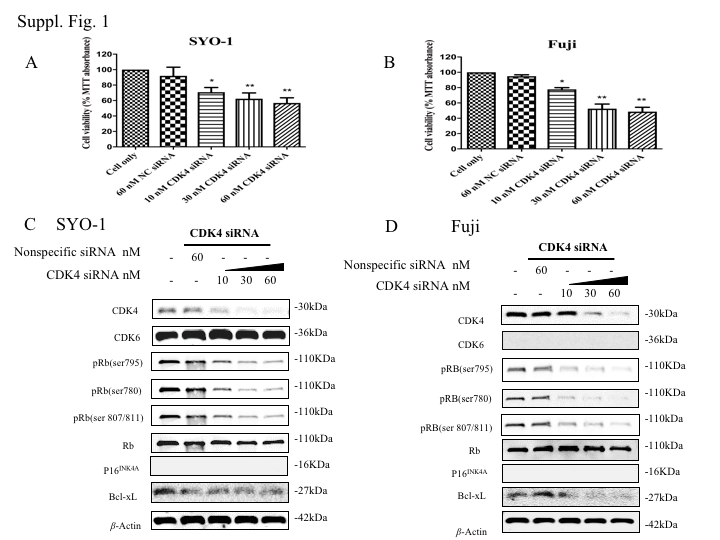

Supplement: Supplementary file 1 — Suppl. Fig. 1(TIF 1522 kb) [file 41419_2018_474_MOESM1_ESM.tif]

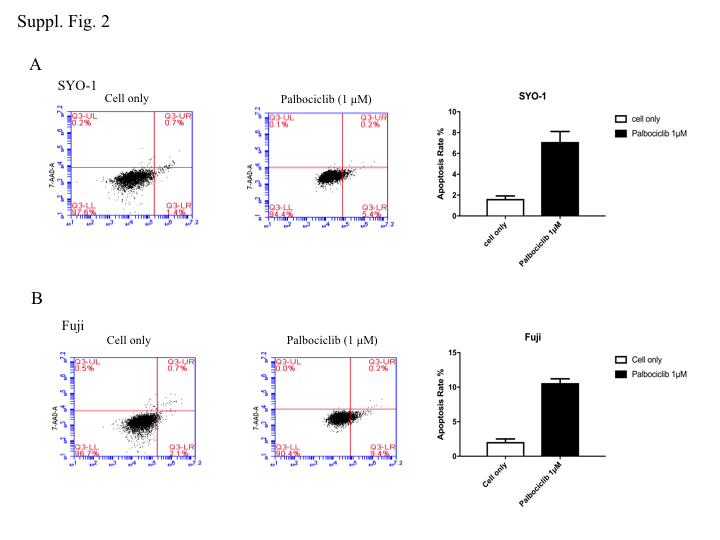

Supplement: Supplementary file 2 — Suppl. Fig. 2(TIF 1522 kb) [file 41419_2018_474_MOESM2_ESM.tif]
