# Supplementary material for: Inhibition of cyclin-dependent kinase 4 as a potential therapeutic strategy for treatment of synovial sarcoma
Source: Cell Death Dis. 2018 Apr 18;9(5):446. doi: 10.1038/s41419-018-0474-4 (PMC5906661; doi:10.1038/s41419-018-0474-4)
Supplement: Supplementary file 3 — Suppl. Table 1(DOCX 70 kb) [file 41419_2018_474_MOESM3_ESM.docx]

**Supplementary Table 1**

**Suppl. Table 1: The relationship between CDK4 expression and clinicopathological features of synovial sarcoma**

| Clinicopathological Features | Number of cases | CDK4 Expression Low | CDK4 Expression High | *P* value |
| --- | --- | --- | --- | --- |
|  | (n, %) | (n, %) | (n, %) |  |
|  |  |  |  |  |
| **All patients** | 50(100) | 26(52.0) | 24(48.0) |  |
|  |  |  |  |  |
| **Age (Year)** | 41.62±15.59 | 41.85±14.20 | 41.38±17.28 | 0.916 |
| ≤40 | 27(54.0) | 14(28.0) | 13(26.0) | 1.000 |
| >40 | 23(46.0) | 12(24.0) | 11(22.0) |  |
|  |  |  |  |  |
| **Gender** |  |  |  |  |
| Male | 30(60.0) | 14(28.0) | 16(32.0) | 0.525 |
| Female | 20(40.0) | 12(24.0) | 8(16.0) |  |
|  |  |  |  |  |
| **Tissue Type** |  |  |  |  |
| Tendon sheath | 30(60.0) | 19(38.0) | 11(22.0) | 0.333 |
| Articulation | 3(6.0) | 1(2.0) | 2(4.0) |  |
| Soft tissues | 4(8.0) | 4(8.0) | 0(0.0) |  |
| Fibrous tissue | 6(12.0) | 0(0.0) | 6(12.0) |  |
| Other | 7(14.0) | 2(4.0) | 5(10.0) |  |
|  |  |  |  |  |
| **Tumor Location** |  |  |  |  |
| Extremities | 42(84.0) | 21(42.0) | 21(42.0) | 0.793 |
| Elsewhere Location | 8(16.0) | 5(10.0) | 3(6.0) |  |
|  |  |  |  |  |
| **Clinical Stage** |  |  |  |  |
| ≥ⅡB | 32(64.0) | 10(20.0) | 22(44.0) | 0.000* |
| <ⅡB | 18(36.0) | 16(32.0) | 2(4.0) |  |
|  |  |  |  |  |
| **TNM Grade** |  |  |  |  |
| ≥G2 | 37(74.0) | 15(30.0) | 22(44.0) | 0.016* |
| <G2 | 13(26.0) | 11(22.0) | 2(4.0) |  |
