# Supplementary material for: Inhibition of cyclin-dependent kinase 4 as a potential therapeutic strategy for treatment of synovial sarcoma
Source: Cell Death Dis. 2018 Apr 18;9(5):446. doi: 10.1038/s41419-018-0474-4 (PMC5906661; doi:10.1038/s41419-018-0474-4)
Supplement: Supplementary file 4 — Suppl. Table 2(DOCX 79 kb) [file 41419_2018_474_MOESM4_ESM.docx]

**Supplementary Table 2**

**Suppl. Table 2: The relationship between CDK4 expression and clinicopathological features of various sarcomas**

| Clinicopathological Features | Number of cases | CDK4 Expression Low | CDK4 Expression High | *P* value |
| --- | --- | --- | --- | --- |
|  | (n, %) | (n, %) | (n, %) |  |
| **All patients** | 59(100) | 31(52.5) | 28(47.5) |  |
|  |  |  |  |  |
| **Age (Year)** | 46.25±18.14 | 43.35±18.36 | 49.46±17.66 | 0.199 |
| ≤40 | 22(37.3) | 14(23.7) | 8(13.6) | 0.295 |
| >40 | 37(62.7) | 17(28.8) | 20(33.9) |  |
|  |  |  |  |  |
| **Gender** |  |  |  |  |
| Male | 31(52.5) | 17(28.8) | 14(23.7) | 0.912 |
| Female | 28(47.5) | 14(23.8) | 14(23.7) |  |
|  |  |  |  |  |
| **Sarcoma Type** |  |  |  |  |
| Synovial sarcoma | 8(13.56) | 7(11.86) | 1(1.70) | 0.324 |
| Osteosarcoma | 2(3.39) | 1(1.69) | 1(1.70) |  |
| Liposarcoma | 7(11.86) | 4(6.78) | 3(5.08) |  |
| Chordoma | 3(5.08) | 2(3.39) | 1(1.69) |  |
| Leiomyosarcoma | 9(15.25) | 4(6.78) | 5(8.47) |  |
| Malignant fibrous histiocytoma | 12(20.33) | 5(8.47) | 7(11.86) |  |
| Rhabdomyosarcoma | 4(6.78) | 1(1.70) | 3(5.08) |  |
| Fibrosarcoma | 2(3.39) | 0(0.00) | 2(3.39) |  |
| Neuronal sarcoma | 6(10.17) | 4(6.78) | 2(3.39) |  |
| Other | 6(10.17) | 3(5.08) | 3(5.09) |  |
|  |  |  |  |  |
| **Tumor Location** |  |  |  |  |
| Extremities | 26(44.07) | 16(27.12) | 10(16.95) | 0.370 |
| Trunk Wall | 14(23.73) | 5(8.47) | 9(15.25) |  |
| Head/Neck | 4(6.78) | 1(1.70) | 3(5.08) |  |
| Abdomen/Retroperitoneum | 11(18.64) | 7(11.86) | 4((6.78) |  |
| Elsewhere Location | 4(6.78) | 2(3.39) | 2(3.39) |  |
|  |  |  |  |  |
| **Prognosis** |  |  |  |  |
| Survival | 31(52.54) | 22(37.29) | 9(15.25) | 0.003* |
| Non-survival | 28(47.46) | 9(15.25) | 19(32.20) |  |
