# Supplementary material for: Inhibition of cyclin-dependent kinase 4 as a potential therapeutic strategy for treatment of synovial sarcoma
Source: Cell Death Dis. 2018 Apr 18;9(5):446. doi: 10.1038/s41419-018-0474-4 (PMC5906661; doi:10.1038/s41419-018-0474-4)
Supplement: Supplementary file 5 — Supplementary figure legends(DOCX 79 kb) [file 41419_2018_474_MOESM5_ESM.docx]

**Supplementary Figure 1.** **CDK4/6-Rb pathway inhibition caused by CDK4 specific siRNA (#SASI_Hs01_00122490) reduces human synovial sarcoma cell proliferation and induces cell apoptosis *in vitro*.** Human synovial sarcoma SYO-1 and Fuji cells were transfected with increasing concentrations of CDK4 specific siRNA (#SASI_Hs01_00122490) or nonspecific siRNA, and cell proliferation and growth was determined subsequently. (A and B) Cell viability was determined by MTT assay after siRNA transfection for 5 days. (C and D) The respective proteins of CDK4/6-Rb-apoptosis pathway in cells were examined by Western blotting after 48 hours of siRNA transfection. ******P* < 0.05, *******P* < 0.01 compared with the cell only group.

**Supplementary Figure 2.** **Inhibition of the CDK4/6-Rb pathway by palbociclib enhances apoptosis in human synovial sarcoma.**, Cell apoptosis rate was analyzed respectively after exposure to palbociclib (1 µM) for 24 hours in SYO-1 (A) and Fuji (B) cells by flow cytometry analysis. Representative images of cell apoptosis in with or without palbociclib treatment were assessed.
